# Metformin Affects Gut Microbiota Composition and Diversity Associated with Amelioration of Dextran Sulfate Sodium-Induced Colitis in Mice

**DOI:** 10.3389/fphar.2021.640347

**Published:** 2021-05-21

**Authors:** Zhiyi Liu, Wangdi Liao, Zihan Zhang, Ruipu Sun, Yunfei Luo, Qiongfeng Chen, Xin Li, Ruiling Lu, Ying Ying

**Affiliations:** ^1^ Jiangxi Institute of Respiratory Disease, The First Affiliated Hospital of Nanchang University, Nanchang, China; ^2^ Queen Mary School, Nanchang University, Nanchang, China; ^3^ Departments of Gastroenterology, The First Affiliated Hospital of Nanchang University, Nanchang, China; ^4^ Department of Pathophysiology, Schools of Basic Medical Sciences, Nanchang University, Nanchang, China; ^5^ The Department of Respiratory and Critical Care Medicine, The First Affiliated Hospital of Nanchang University, Nanchang, China

**Keywords:** inflammatory bowel disease, metformin, gut microbiota, anti-inflammatory effect, biodiversity

## Abstract

**Background:** Inflammatory bowel disease (IBD) is an increasingly common and globally emergent immune-mediated disorder. The etiology of IBD is complex, involving multiple factors such as immune dysregulation, environmental factors, genetic mutations, and microbiota dysbiosis, exacerbated by a lack of effective clinical therapies. Recently, studies hypothesized that dysbiosis of intestinal flora might participate in the onset of IBD. Metformin is widely used to treat type 2 diabetes and has shown beneficial effects in mouse models of IBD, although its underlying mechanisms remain poorly understood. Accumulating studies found that metformin shows beneficial effects for diabetes by affecting microbiota composition. This study explores possible regulatory effects of metformin on intestinal microecology during treatment for IBD.

**Methods:** Inflammation was induced using 3% Dextran Sulfate Sodium (DSS) solution to generate mice models of IBD. Metformin treatments were assayed by measuring body weights and colon lengths of mice and H&E staining to observe histological effects on colon tissue structures. Changes in bacterial community composition and diversity-related to IBD and metformin treatment were assessed by high-throughput metagenomic sequencing analysis.

**Results:** Metformin administration significantly ameliorated body weight loss, inhibited colon shrinking, and contributed to preserving the integrity of colon histological structures. The gut microbiota profiles revealed that the biodiversity of intestinal flora lost during inflammation was restored under metformin treatment. Metformin administration was also associated with decreased pathogenic *Escherichia shigella* and increased abundance of *Lactobacillus* and *Akkermansia*.

**Conclusion:** Metformin appears to induce anti-inflammatory effects, thus ameliorating colitis symptoms, concurrent with enrichment for beneficial taxa and restored microbial diversity, suggesting a viable strategy against IBD.

## Introduction

Inflammatory bowel disease (IBD), characterized by chronic inflammatory disorders of the colon and small intestine, are common and widespread. Ulcerative colitis (UC) and Crohn’s disease (CD) are the two main types of IBD, manifested with severe intestinal disorders, including diarrhea, abdominal pain, weight loss, and bloody stools (De Souza and Fiocchi, 2016). The IBD-associated immune reaction is triggered by intestinal components, especially gut microbiota, which are suspected of contributing to susceptibility to CD and UC (Ni et al., 2017). Evidence has been reported in both human and animal studies for a potential role for imbalance among gut microbiota in the pathogenic mechanisms of IBD (Matsuoka and Kanai, 2015; Nishida et al., 2018). According to clinical observations, the majority of colitis lesions in IBD patients are located in segments where gut microbes are highly concentrated, significantly associated with fecal accumulation, such as the colorectal terminal segment (Ni et al., 2017). Molecular and high-throughput sequencing analysis of gut microbiota in IBD patients has revealed changes in characteristic microbial profiles, or dysbiosis, characterized by disruption in the balance between commensal, intestinal probiotics, and opportunistic pathogens, ultimately resulting in decreased gut biodiversity (Wlodarska et al., 2015).

Metformin is a widely used drug for the treatment of type 2 diabetes, and its therapeutic mechanisms are associated with the activation of 5′-adenosine mono-phosphate-activated protein kinase (AMPK) to reduce insulin resistance. AMPK is also involved in anti-inflammatory pathways by inhibiting pro-inflammatory cytokines production (Kim et al., 2014), which suggests a therapeutic role for metformin in inflammatory disease and immune disorders. Furthermore, in diabetic and high-fat diet (HFD) rats, enrichment for a beneficial intestinal bacterial community was observed with long-term metformin treatment. The abundance of short-chain fatty acid-producing bacteria increased significantly, and the level of *Lactobacillus*, *Prevotella*, and A*kkermansia muciniphila* was also elevated, thus improving the maintenance of metabolic processes and the homeostasis of intestinal tissue (Bauer et al., 2018).

Here, we explored the intervention effects of metformin on dysbiosis of intestinal microecology in DSS-induced colitis mice model. To this end, we histologically evaluated the degree of inflammation in intestinal tissue structures, in addition to assessing overall changes in body weight and colon length under metformin pretreatment, treatment and non-treatment conditions during DSS-induced colitis. We also profiled gut microbiota via high-throughput sequencing of the v3-v4 region of 16s ribosomal DNA (rDNA) for metagenomic analysis of microbiota composition and diversity. We report restoration of diversity under metformin treatment that was lost under inflammatory conditions and enrichment for beneficial taxa associated with ameliorated colitis symptoms following metformin administration. This work provides insight into the relationship between metformin exposure and gut microbiota in an induced mouse model of intestinal colitis and suggests a potentially viable clinical strategy for this widespread disease.

## Materials and Methods

### Experimental Animals

Forty C57BL/6 male mice, aged 8–10 weeks, were purchased from the laboratory animal center of Nanchang University. The mice were housed in a sterile, ventilated room for a week at 20–25°C and given sufficient sterile food and water. All the animal protocols were approved and followed the guidelines and laws of the Animal Care Committee of Nanchang University Jiangxi Medical College (Animal protocol: NCDXSYDWFL-2015097).

### Instruments and Reagents

Dextran Sodium sulfate powder (Dextran Sulfate Sodium, DSS, MW 36,000–50,000) was purchased from the MP Biomedicals company (United States). Metformin was from Sigma (Germany). TransGen AP221-02:TransStart Fastpfu DNA Polymerase (TransGen Biotech, Beijing, China), AxyPrep DNA gel extraction kit (AXYGE, United States), QuantiFluorTM-ST (Promega, United States), TruSeqTM DNA Sample Prep Kit (Illumina, United States).

A bx-41 binocular optical microscope was purchased from Olympus company (Japan), polymerase chain reaction instrument (ABI GeneAmp® 9700, ABI, United States), Embedding cassettes (Citotest Labware Manufacturing Co., Ltd., China), KD-TS3D tissue processor (KEDEE, United States).

### Induction of Experimental Intestinal Bowel Disease

The procedures of disease induction were carried out as follows. The C57BL/6 male mice were given sterile drinking water for acclimation 7 days before induction of colitis. Then forty mice were randomly divided into five groups (as shown in Table 1): 1) control group (C group) receiving sterile drinking water and PBS intraperitoneal injection. 2) DSS-induced colitis model group (D group) receiving PBS intraperitoneal injection. 3) metformin treatment for DSS-induced colitis (MD group) receiving metformin intraperitoneal injection (150 mg/kg/day) 3 days after disease induction. 4) metformin pretreatment for DSS-induced colitis group (P group) receiving metformin injection (150 mg/kg/day) one day before disease induction. 5) metformin group (M group) receiving sterile water and intraperitoneal injection of metformin (150 mg/kg/day). The weight of each mouse was measured daily at the same time. At eight days after giving 3% DSS solution, the mice were sacrificed, and the length of the colons above 5 mm from the end of the rectum was measured and recorded. After rinsed by PBS solution, the colons were divided into the upper, middle, and lower segments, and the middle parts were collected. The feces samples were rapidly collected from the end of the colon and stored in −80°C refrigerator.

**TABLE 1:** Grouping and time of medication.

Groups\Time(days)	Day 0	Day 1∼3	Day 4∼7
C group	PBS injection	Sterile water + PBS injection	Sterile water + PBS injection
D group	PBS injection	DSS solution + PBS injection	DSS solution + PBS injection
MD group	PBS injection	DSS solution + PBS injection	DSS solution + metfromin injection
P group	Metformin injection	DSS solution + metformin injection	DSS solution + metformin injection
M group	PBS injection	Sterile water + metformin injection	Sterile water + metformin injection

Note: C = Control, D = DSS group, MD = DSS + Metformin, P = DSS + pre-Metformin, M = Metformin.

### Histopathological Analysis

The colon tissues were fixed in 4% formalin solution for 24 h and then washed thoroughly with PBS solution, dehydrated, and embedded in paraffin. The tissues were cut into 4 µm slices by lycra slicer and stained with H&E stain. Finally, the tissue sections were observed and photographed at 100x and 400x magnification with an optical microscope.

### Gut Microbiota Analysis

The feces samples kept in dry ice was sent to Shanghai Majorbio Bio-pharm Technology Co., Ltd., for DNA extraction and sequencing. The gut microbiota DNA was extracted from lumenal stools, and PCR reactions amplified the fragments of 16s rDNA v3-v4 region with TransStart Fastpfu DNA Polymerase (AP221-02), and the final products were quantified by QuantiFluorTM-ST (Promega, Beijing, China). The DNA was collected by AxyPrep DNA gel extraction kit (AxyPrep Co., Ltd.). Then, the purified DNA products were processed by TruSeqTM DNA Sample Prep Kit and sequenced by Illumina Miseq. The sequencing data were spliced, quality controlled to obtain an optimized sequence with FLASH (v1.2.11). Moreover, subsequently, the bacterial sequences were clustered by Uparse (v7.0.1090) on the level of OTU (Operational Taxonomic Units) that classifies bacteria with 97% similarity of 16 s gene sequences. The OTU abundance table was conducted for subsequent biogenetic analysis.

The alpha diversity of observed OTU level and Shannon index were analyzed on the platform of the Majorbio cloud. Beta diversity analysis based on the Bray Curtis distance and Weighted unifrac algorithm was presented by the Principal coordinate analysis (PCoA) figure, and Adonis calculated the difference between groups. The linear discriminant analysis effect size (LEfSe) analysis was performed to represent the biomarkers of samples on different taxonomic levels using the LDS score to estimate the influence of richness on the extent of the difference.

### Statistical Analysis

The data and statistical analysis were conducted by GraphPad Prism (v.8.2.1). The results of the quantitative analysis were presented by mean ± standard error of the mean (SEM). The significant differences were evaluated either by unpaired Student’s *t*-test, two-way analysis of variance and by Wilcoxon rank-sum method or Mann-Whitney U test for non-parametric data. *p* < 0.05 indicates statistical significance. Asterisks used to indicate significance correspond with: *p < 0.05, **p < 0.01, ***p < 0.001.

## Results

### Metformin Ameliorates General Symptoms and Histopathological Change in DSS-Induced Colitis Mice

**FIGURE 1:**
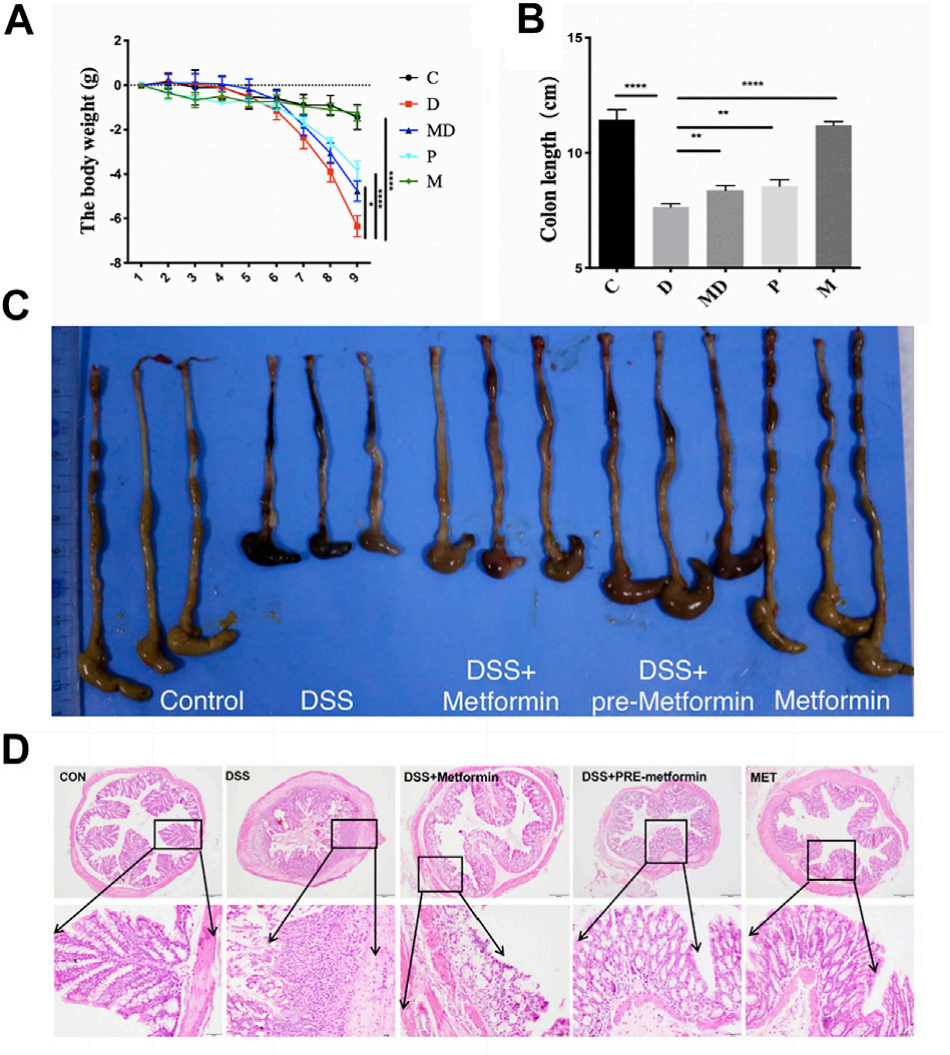
Metformin prevents DSS-induced colitis in mice. C57BL/6 mice were divided into five groups and induced acute colitis by giving 3% DSS. The mice were treated with intraperitoneal PBS or metformin (150 mg/kg/day) daily. **(A)** The body weight changes during the colitis induction process. Significance was tested by Two-way ANOVA. *p < 0.05 ****p < 0.0001, *n* = 8. **(B)** The colon length of each group. The Significance was analyzed by student t-test (mean ± SEM, *n* = 8), **p < 0.01, ***p < 0.001. **(C)** The representative macroscopic image of mice colon. **(D)** Histology analyzed the severity of colitis in DSS with H&E staining. Representative images are shown. Overview: 100 ×; magnification: 400 ×, Scale bar = 100 µm and 20 µm.

Prior to investigating a potential relationship between metformin and microbiota in the amelioration of UC and IBD, we first examined the effects of metformin treatment on C57BL/six mouse body weight and colon length. The weights of individual mice were measured daily during the colitis induction period (Figure 1A). The body weights of the control (C) and metformin-treated healthy mice (M) similarly showed a slight but non-significant decrease during the first two days of treatment but remained stable over the remaining period of model establishment. In contrast, the DSS-induced colitis (D) and DSS-induced colitis with metformin treatment (MD) groups did not show any apparent weight loss until the third day, at which point the D group exhibited a dramatic weight reduction following DSS administration, while the MD group showed significant alleviation of colitis-associated weight loss (*p* < 0.05). The mice that received metformin pretreatment (P) showed slight weight loss but had delayed occurrence and a smaller range of weight loss compared with the D group (*p* < 0.0001).

These changes in body weight due to DSS were reflected by a marked decrease in colon lengths of induced colitis mice (Figure 1B). The colons of the C and M mice were the longest among these five groups, significantly longer than those of the D group (*p* < 0.0001). In addition, metformin treatment (M) and pretreatment (*p*) significantly inhibited colon shortening compared with untreated colitis (D) mice (*p* < 0.01) but did not show complete restoration of the colon length to that of non-colitis mice. These results show that metformin administration can counteract colitis’s negative impacts on body weight and colon length in DSS-induced colitis mice.

To better understand the changes associated with colitis and metformin treatment at the cellular level in colon tissue, we conducted a histological evaluation of each treatment group (Figure 1D). To this end, colon sections from each of the five groups were sampled at the eighth day after giving DSS solution, processed by H&E staining, and observed by light microscopy. At 100X magnification, we observed that the intestinal layer and glands of the D group exhibited substantially greater inflammatory cell infiltration in the mucosal epithelium and lamina propria, loss of glandular parts, and hypertrophy of the colon wall compared to that of C and M groups. In the MD and *p* groups, the muscular layers were more intact, and the arrangement of intestinal glands appeared relatively healthy. Under 400X magnification, we found that the mucosal columnar epithelium and goblet cells in the gut glands were severely damaged, and the architectures of both crypt and villus were disrupted in the colons of D group mice. In sharp contrast with MD and *p* groups after metformin treatment, the inflammatory cells were significantly decreased, and more intact mucosal epithelium was observed. Moreover, more significant alleviation of inflammation was observed among P group mice, with a large number of goblet cells and longer, well-organized glands. These results together indicate that metformin protects the histological architecture of the colon and ameliorate pathological damages during the process of colitis induction.

### Metformin Restores Biodiversity of Gut Microbiota in Induced-Colitis Mice

**FIGURE 2:**
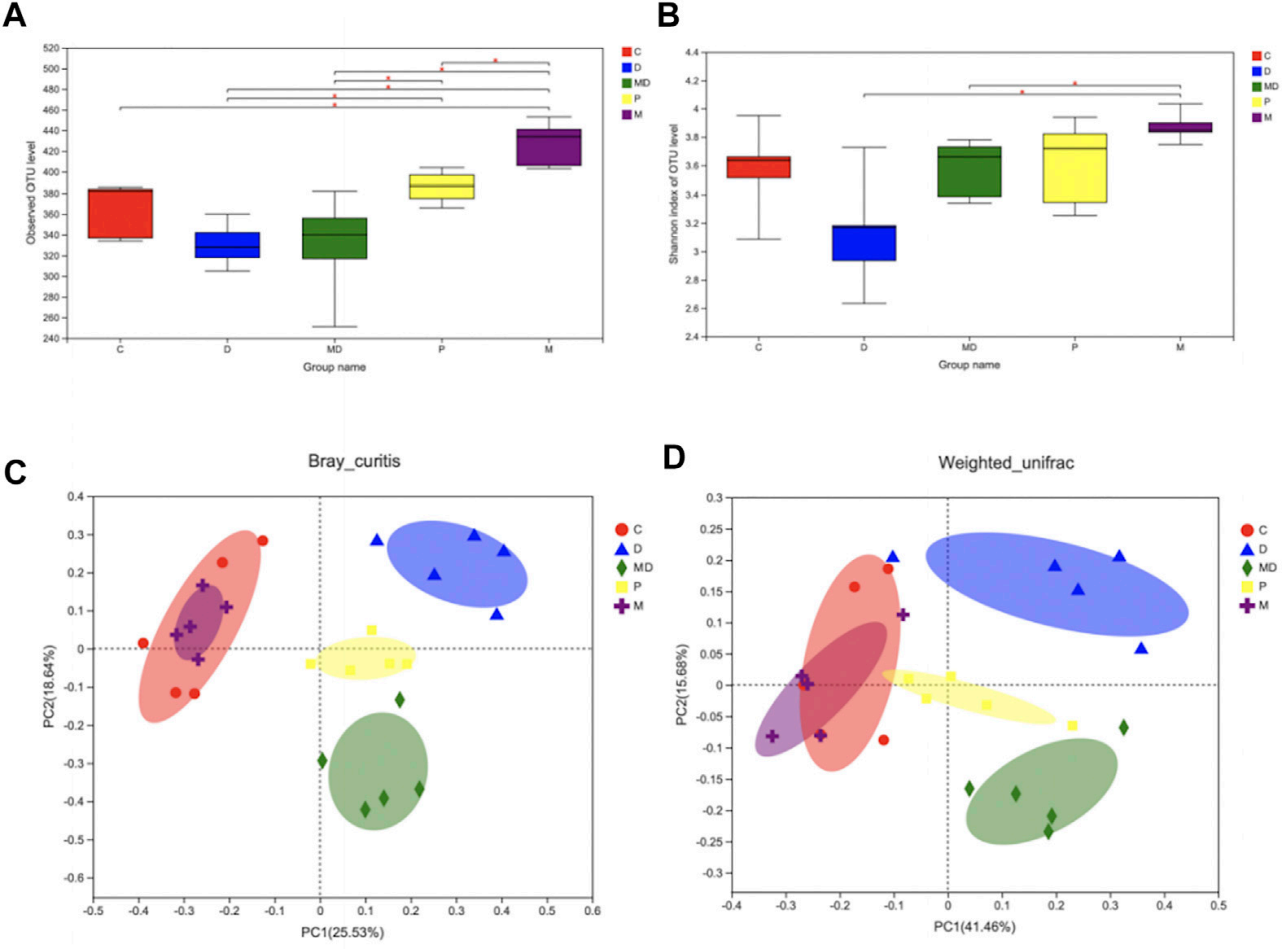
Metformin restores the species diversity and richness of gut microbiota in DSS-induced colitis. **(A)** The Alpha-diversity index is based on observe-OTU of intestinal flora within each group. **(B)** The Alpha-diversity index is based on Shannon of intestinal flora within each group. **(C)** Beta-diversity (PCoA analysis based on Bray_curitis). **(D)** Beta-diversity (PCoA analysis based on Weighted_unifrac). Significance was tested by Wilcoxon rank-sum method. (Data are presented as mean ± SEM.*p < 0.05. C = Control, D = DSS + PBS injection, MD = DSS + Metformin injection, P = pre-Metformin injection, M = only Metformin injection).

In light of the apparent damage associated with colitis and the effects of metformin treatment, we evaluated differences in gut microbial diversity among the treatment groups. The results of observed-OTU index analysis showed that the M group had the highest diversity of OTUs, which was significantly greater than that of the other four groups (*p* < 0.05) (Figure 2A). However, there was no significant difference in either the observed-OTU or Shannon Alpha diversity indexes between the MD group and D groups (Figures 2A,B), although the trend indicated increasing diversity of intestinal flora after metformin treatment. Meanwhile, *p* group mice showed considerable restoration of bacterial diversity with longer metformin treatment courses and were significantly different from the D group (*p* < 0.05) (Figure 2A).

The results of OTU-level principal component analysis (PCA) revealed differences in Beta diversity, an indicator of group similarity calculated by the Bray_Curtis distance and Weighted_unifrac algorithms, among these groups (Figures 2D,C). The C and M groups shared a large overlapping area that suggested a large number of bacteria were shared between these two groups, whereas the biological composition of gut microbiota was significantly changed in the D group. Clustering patterns of the MD and P groups revealed that they became more similar to the C group following metformin treatment, while the difference between the P and C groups was smaller than that between the MD and C groups. These results indicate that metformin treatment does not affect healthy microbiota diversity but can restore the diversity of mice with induced colitis.

### Metformin Restores Gut Microbiota Composition of Induced-Colitis Colons

**FIGURE 3:**
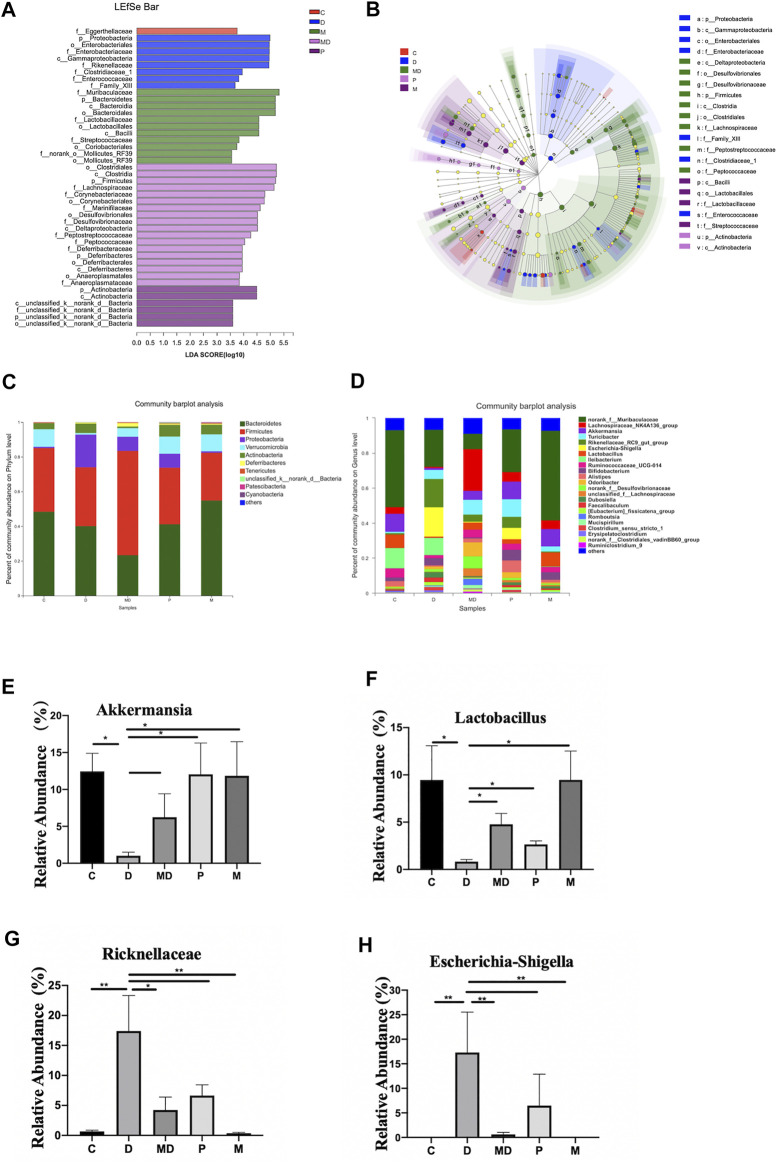
Metformin exerts regulatory effects on the balance of some inflammation-related bacteria proportion and the overall composition of the community. **(A)** The bar plot of differential microbiota distribution among different groups based on LDA value. **(B)** The cladogram of differential microbiota on the level from Phylum to Family among different groups. **(C)** The bar plot of community composition on the Phylum level. **(D)** The bar plot of community composition on the Genus level. **(E)** The relative abundance of *Escherichia shigella* **(F)** *Lactobacillus* **(G)** *Akkermansia* **(H)** *Ricknellaceae* within each group. The *p*-value was calculated by the Mann-Whitney *U* test. *p < 0.05, **p < 0.01. C = Control, D = DSS group. MD = DSS + Metformin, P = DSS + pre-Metformin, M = Metformin.

We then conducted LEfSe analysis to identify differentially enriched microbiota within each group that could potentially serve as biomarkers for colitis in the gut. This analysis showed that at the phylum level, the M group was significantly associated with *Bacteroidetes*, D group with *Proteobacteria*, MD group with *Firmicutes*, and the P group with *Actinobacteria* (Figures 3A,B). Analysis of phylum-level (other <0.01) community composition showed that *Bacteroidetes*, *Firmicutes*, *Proteobacteria*, *Verrucomicrobia*, and *Actinobacteria* accounted for the largest proportions of phyla in all groups (Figure 3C). Community barplot analysis of genus-level (other <0.01) also showed that *Escherichia-Shigella*, *Lactobacillus,* and *Akkermansia* and *Ricknellaceae* taxa which are potentially associated with IBD pathogenesis and therapeutic effects of metformin, were varied among D, MD, and P groups (Figure 3D). Further analysis of the relative abundance of individual taxa revealed that the C and M groups shared a similar proportion of these four bacteria (Figures 3E–H). However, compared with the C group, the D group showed a significant increase in *Escherichia-Shigell*a, although the growth of this genus was inhibited in the MD and P groups. The relative abundances of *Lactobacillus*, *Akkermansia, and Ricknellaceae* were significantly reduced in the D group compared with control mice but exhibited a substantial increase after treatment with metformin. The pretreatment group also showed significantly higher levels of *Lactobacillus, Akkermansia, and Ricknellaceae* than those of the D group (*p* < 0.01). These results together suggest that disruption of colon community composition by colitis, resulting in over-representation of disease-associated taxa, can be restored by metformin treatment to a similar composition as that of a healthy colon environment.

## Discussion

In this study, we examined the potential inflammatory disease therapeutic metformin for its effects on diversity and composition of colon microbiota in a DSS-induced mouse model of IBD. We confirmed that induced colitis disrupted the architecture of the intestinal epithelium and subsequently enriched the guts of these mice for bacterial taxa associated with inflammatory disease. Our results showed that treatment with metformin did not disrupt the microbiota composition or diversity of healthy mice. Moreover, metformin administration, either before or after the induction of colitis, resulted in amelioration of physical symptoms and also partially restored microbiota composition and diversity to more closely resemble that of healthy, non-colitis mice. Notably, we identified several enriched phyla that were indicative of specific treatments or disease states. Most notably, we observed the differential prevalence of genera that were significantly associated with disease, such as *Escherichia* sp., which were absent from healthy mice but enriched in colitic mice, *Lactobacillus*, which were prevalent in healthy and metformin-treated mice, and *Akkermansia*, which was absent in colitic mice but restored in metformin-treated mice. We also found high amounts of *Ricknellaceae*, which were specifically enriched during and after colitis, and *Turicibacter*, which were absent from the healthy controls. Our findings provide useful insights into the effects of metformin on enrichment for gut bacteria associated with healthy colon conditions during treatment for IBD.

Phenotypic examination of the induced-colitis mice confirmed that body weight and colon length were both reduced by colitis and that intestinal glands had severe infiltration by inflammatory cells infiltration, disrupted intestinal crypts, and depletion of goblet cells. These symptoms of dysbiosis in the gut microenvironment have been previously associated with pathogenic taxa (Ni et al., 2017), which are both enriched by inflammatory conditions, and also contribute to the disease progression. Intestinal microbiota has been shown to participate in interaction networks resulting in the formation of a protective barrier that can suppress excessive immune responses and ensure the integrity of the intestinal tract (Wu et al., 2020). Previous work in a mouse model of IBD has shown that metformin administration can attenuate inflammation and exert protective effects on the intestinal barrier (Lee et al., 2015). Moreover, a recent longitudinal cohort study found that long-term use of metformin provided prophylactic effects against IBD in type 2 diabetes mellitus patients (Tseng, 2020). Metformin may potentially preserve the community composition of healthy state gut microbiota through proposed anti-inflammatory mechanisms, including inhibition of pro-inflammatory cytokines production (Xue et al., 2016), activation of AMPK (Bułdak et al., 2016), improved insulin resistance (Viollet et al., 2012), or up-regulation of postbiotic (short-chain fatty acid and vitamin D) production. However, much further investigation is required to establish a causative relationship between metformin administration and restoration of healthy microbiota in IBD patients.

We next focused on the beneficial effects of metformin on the diversity of intestinal flora. The alpha diversity index at the OTU level indicated that metformin treatment leads to increased richness and diversity of gut microbiota, which ameliorated the dominance of fewer, inflammation-associated taxa, and hence the low bacterial biodiversity of colitic mice. We observed an increasing trend but no significant difference in alpha diversity between the D and MD groups, and this might indicate that the influence of metformin to restore gut biodiversity may also rely on other contributing factors, such as treatment time, diet. For example, our diversity analysis of the P group showed that a longer treatment course or a prophylactic treatment with metformin facilitated the recovery of biodiversity. This finding is consistent with that reported by Zhang and colleagues, who found metformin effect to increase microbial diversity in diabetic mice (Zhang et al., 2019). Nevertheless, mice fed with a high-fat diet exhibited had reduced microbial diversity after metformin administration, whereas mice fed with chow and treated with metformin did not experience a significant change in gut biodiversity (Zhang et al., 2015)*.* One possible explanation for these variations in the effects of metformin is that metformin can ostensibly shift microbial diversity toward ecological equilibrium, as proposed by Elbert and co-workers, but in a manner dependent on other factors such as disease or diet since metformin did not appear to shift the diversity of healthy communities (Elbere et al., 2018).

Several specific pathogens reported to induce or exacerbate IBD-associated diseases, e.g., *Mycobacterium paratuberculosis* (Crohn’s disease) (Zarei-Kordshouli et al., 2019) or *Fusobacterium nucleatum (ulcerative colitis)* (Chen et al., 2020), are likely to outcompete and subsequently reduce the abundance of beneficial microbiota that bind to epithelial receptors for survival, such as *Lactobacillus* and butyrate-producing bacteria (e.g., *Faecalibacterium prausnitzii*) (Takaishi et al., 2008; Eppinga et al., 2016). Under healthy conditions, these beneficial taxa help stabilize intestinal flora by inhibiting pathogen colonization, promoting mucus production, and enhancing immune response (Wlodarska et al., 2015). We observed the loss of several potentially beneficial taxa, as well as enrichment for other poorly understood, and potentially pathogenic OTUs, from colitic mice. At the phylum level, IBD patients have characteristically reduced ratios of obligate anaerobes such as *Firmicute* and *Bacteroidetes*, but enriched levels of facultative anaerobes, especially *Proteobacteria* and *Actinobacteria* (Matsuoka and Kanai, 2015). Consistent with these prior studies, we observed a significantly higher abundance of *Proteobacteria*, especially *Escherichia-Shigella* associated with the untreated (D) colitis group, which has been correlated with relapse of Crohn's disease in other studies (Bai and Ouyang, 2006; Cantó et al., 2019). However, we also observed increased *Firmicute abundance* in the treated (MD) colitis group, strongly suggesting that metformin may help restore taxa associated with health conditions. However, whether and how metformin promotes colonization of specific taxa requires much more in-depth investigation.

In this study, we also found that the relative abundances of few probiotics were significantly enriched in response to metformin treatment (and especially in the pretreatment group), such as that of *Akkermansia,* which has been reported by similar previous studies (Guo et al., 2017). This commensal species reportedly inhibits enteric disease development by maintaining the integrity of the mucosal barrier through mucin degradation, which leads to the freshening of the mucosa, prevention of pathogen establishment, and a clean environment conducive to physiological function (Everard et al., 2013). Moreover, *Akkermansia* administration also reportedly contributed to the prevention of weight loss and inflammatory responses associated with IBD (Bian et al., 2019). Similarly, *Lactobacillus* sp. abundance decreased in the diseased group but was restored after metformin treatment, which was consistent with the previously observed effect of metformin (Zhang et al., 2019). However, in contrast with *Akkermansia*, pretreatment had no apparent positive effects on *Lactobacillus* abundance compared with direct treatment. Interestingly, we found that *Ricknellaceae* abundance increased after metformin treatment, consistent with fluctuations related to *Ilex kudingcha* and 2-O-β-D-glucopyranosyl-L-ascorbic acid treatment of DSS-induced colitis. (Huang et al., 2019; Wan et al., 2019), although its relationship with colitis requires further investigation.

In marked contrast with these non-pathogens, *Escherichia shigella*, a common, opportunistic, causative agent for diarrhea(Kotloff et al., 2013), was suppressed after metformin administration. *Escherichia shigella* may enhance the side effects of metformin, and 20–30% of patients with long-term metformin treatment had moderately increased abundance of *E. shigella* abundance, associated with mild diarrhea, in agreement with its enrichment in pretreated mice that had longer metformin courses (Elbere et al., 2018). However, we found that *E. shigella* was not significantly enriched in metformin-treated healthy mice, nor did these mice display diarrhea. In fact, E. *shigella* abundance decreased in diseased mice following metformin treatment, indicating that the relationship between metformin and this pathogen warrants closer scrutiny, perhaps under isolated, *in vitro* conditions.

## Conclusion

In conclusion, our study showed the effects of metformin on intestinal flora during treatment for IBD, especially highlighting the restoration of microbial diversity and enrichment in the relative abundance of specific taxa. These effects on gut microbiota appear to be related to anti-inflammatory effects required for the alleviation of the symptom of DSS-induced colitis. Therefore, we propose that metformin is a strong potential candidate for IBD therapy, and further investigation of metformin-induced changes in gut microbiota and its anti-inflammatory effects should be investigated to clarify its molecular interactions the gut.

## Data Availability

The data generated in this article can be found here: https://www.ncbi.nlm.nih.gov/sra/PRJNA682001.

## Abbreviations

IBD, inflammatory bowel disease; DSS, dextran sodium sulfate; UC, ulcerative colitis; CD, crohn’s disease; AMPK, 5′-adenosine mono-phosphate-activated protein kinase; H&E, hematoxylin and eosin; C group, control group; D group, DSS-induced colitis model group; MD group, metformin treatment for DSS-induced colitis group; P group, metformin pretreatment for DSS-induced colitis group; M group, metformin group; 16SrRNA, 16S ribosomal RNA; OTU, operational taxonomic unit; PBS, phosphate buffer saline; PCoA, principal coordinates analysis; PCR, polymerase chain reaction; LDA, linear discriminant analysis; LefSe, LDA effect size.
